# Integrating host immune status, *Labyrinthula* spp. load and environmental stress in a seagrass pathosystem: Assessing immune markers and scope of a new qPCR primer set

**DOI:** 10.1371/journal.pone.0230108

**Published:** 2020-03-13

**Authors:** Paige Duffin, Daniel L. Martin, Katrina M. Pagenkopp Lohan, Cliff Ross

**Affiliations:** 1 Department of Biology, University of North Florida, Jacksonville, Florida, United States of America; 2 Smithsonian Environmental Research Center, Edgewater, Maryland, United States of America; Tallinn University of Technology, ESTONIA

## Abstract

Recent trends suggest that marine disease outbreaks caused by opportunistic pathogens are increasing in frequency and severity. One such malady is seagrass wasting disease, caused by pathogens in the genus *Labyrinthula*. It is suspected that pathogenicity is intimately linked to the ability of the host to initiate defense responses; however, supportive evidence is lacking. To address this, we developed two techniques, including 1) a new qPCR-based pathogen detection method, and 2) an immune profiling panel via four host-biomarker assays (measuring peroxidase, exochitinase, polyphenol oxidase, and lysozyme activities). These techniques were then used to experimentally investigate the impact of environmental stressors (namely, elevated temperature and salinity) on host immunity and how immune status might affect susceptibility to *Labyrinthula* infection. In the first experiment, we subjected individual turtlegrass (*Thalassia testudinum*) shoots to short-term (7 d) abiotic stressors alone. In a second experiment, the same abiotic stressor conditions were followed by pathogen exposure (7 additional d), simulating a scenario where we attempt to isolate the impact of environmental stressors on the host seagrass species by removing the stressor as the pathogen is introduced. The qPCR assay successfully quantified the abundance of *Labyrinthula* spp. cells from both pure cultures and seagrass tissues across a broad range of predominately pathogenic strains, with high sensitivity. Immune enzyme assays revealed that all four biomarkers were constitutively active in turtlegrass individuals, but specific activities were largely unaffected by the chosen abiotic stressor conditions. We also identified positive correlations between pathogen load and two biomarkers (peroxidase, exochitinase), regardless of abiotic stress treatment, further demonstrating the potential utility of these biomarkers in future applications.

## Introduction

Marine pathogens can have profound impacts on their hosts, with disease outbreaks leading to sudden population declines in many taxonomic groups [1–4]. One such example is seagrass wasting disease (SWD), which involves complex relationships among marine vascular plants, environmental parameters, and protistan stramenopiles of the family Labyrinthulaceae [5–8]. *Labyrinthula* spp. move within a self-produced ectoplasmic network and degrade host cell walls via extracellular enzymes [8]. Disease symptoms include splotching and dark (brown-to-black) streaking on leaves, which initiate on the leaf epidermis and eventually penetrate internal tissues, leading to defoliation in extreme cases [9]. This disease affects seagrasses from local to regional scales (e.g., 90% loss of north Atlantic *Zostera marina*; reviewed in [10]). Additionally, parasitic varieties can vary in their host-specificity, pathogenicity, and virulence [5, 11], though the specific mechanisms underpinning natural disease events remain elusive.

A growing body of research points to links between climatic changes and an increase in transmission, host susceptibility, and frequency of marine diseases [1, 3–4]. However, a better understanding of host-pathogen interactions in the marine environment, the induced responses mediating them, and the role of the environment in altering their outcome should aid in understanding outbreak dynamics. The apparent opportunistic nature of some *Labyrinthula* spp. [12] implies that pathogenicity involves a delicate interplay between physiological conditions of the host and pathogen, whereby environmental stressors can shift the advantage towards one party over the other [6–7, 10]. Thus, SWD is generally thought to occur when conditions disfavor the seagrass host, resulting in a suppressed or weakened defense system that is susceptible to infection [6–7, 12–13]. However, the hypothesis that abiotic environmental stressors, such as reduced light availability, hypoxia, and elevated salinity, suppress seagrass immunity [6, 14–15] has not been addressed because disease-related metrics of immunity have not been clearly established in seagrasses.

Considering seagrasses are descendants of terrestrial angiosperms that re-entered marine waters ~75 million years ago, it is expected that components of their innate immunity should have a significant degree of overlap [16–17]. Higher plants have evolved a number of immune-related enzymes which may be maintained at constitutive levels or can be induced or activated following pathogen recognition [18–19]. Several common, well-studied enzymes with suspected or well document roles in pathogen defense include peroxidase (POX), polyphenol oxidase (PPO), exochitinase (EXOC) and a group of enzymes with lysozyme-like activity (LYS). POXs are multifunctional redox enzymes [20–21] that regulate biochemical processes such as the cross-linking of cell wall components, the production of secondary metabolites and key reactions in the hypersensitive response [21]. PPOs have been characterized as a common component of the plant innate immune response [22] and are responsible for catalyzing the oxidation of monophenols and/or *o*-diphenols to *o*-diquinones. Through secondary reactions, *o*-diquinones can promote quinone toxicity and confer resistance against pathogens [23–24]. The brown color of quinone adducts that forms as a result of PPO activity may be evident in lesions of infected seagrass tissue. Chitinolytic enzymes, or chitinases, represent a large family of pathogen-related proteins that are responsible for degrading chitin and are strongly induced upon pathogen pressure [25]. EXOCs represent a subcategory of chitinases that catalyze the progressive cleavage of N-acetylglucosamine residues from the non-reducing end of chitin and are noted to have a role in plant disease resistance [26]. Finally, LYS refers to a group of enzymes with lysozyme-like activity and are present in many taxa including plants. They function to hydrolytically cleave components of peptidoglycan, effectively breaking down the cell walls of bacteria [27]. In addition, some plant LYSs have been reported to exhibit antifungal activity against a broad suite of pathogens [28].

Studies assessing seagrass defenses have been chiefly limited to phenolic acid production and other associated secondary metabolites [29–33]. However, in higher plants, such phytochemicals are generally known to play a wide variety of ecological roles, in addition to herbivore and pathogen defense [34–35]. Additionally, Brakel et al. [36] studied changes in gene expression in the eelgrass host *Zostera marina* following nutrient enrichment and/or inoculation with a species of the pathogen known to infect eelgrass (*Labyrinthula zosterae*). They found evidence of upregulation in stress genes like *Hsp80* [36], but this investigation only surveyed expression under conditions of mild virulence, and may not represent defense activity under situations of higher virulence and/or in other seagrass species, like *Thalassia testudinum*.

In an attempt to broaden the suite of possible biomarkers for studies of innate immunity and resistance in seagrasses, we evaluated a series of enzyme-related bioassays which would allow us to better address, for example, whether common immune markers are constitutive in nature and/or induced in response to infection. We also investigated whether there is a link between environmental stressors and immune status, or between environmental stressors and disease susceptibility.

Current methods in SWD quantification may skew our ability to accurately resolve the aforementioned questions. More specifically, the severity of disease in plant tissue has been measured historically using the Wasting Index (WI) [37]. However, there are limitations to this method, including: 1) the pathogen may be present and inflicting damage before lesions are visible [38], and 2) lesions due to *Labyrinthula* spp. infection may be indistinguishable from naturally senescent plant tissue. Bergmann et al. [39] developed a quantitative real-time polymerase chain reaction (qPCR) approach to specifically quantify loading of *L*. *zosterae* (mostly associated with eelgrass); however, as some *Labyrinthula* spp. may co-evolve with their host(s), this assay cannot accurately assess infection by other pathogenic *Labyrinthula* spp. [5, 39]. In order to help address gaps in our understanding of the broader seagrass-*Labyrinthula* pathosystem, we designed a new qPCR assay to include all putatively seagrass-pathogenic *Labyrinthula* spp. (i.e. the “pathogenic” clade, sensu [5]).

*Thalassia testudinum* (turtlegrass) was used as a model host species throughout this study. *T*. *testudinum* is a long-lived seagrass ranging throughout the Gulf of Mexico, the Caribbean, and into parts of the Atlantic; and, often represents the dominant benthic macrofauna in almost all regions where it is found [15, 40]. One of the most significant historical seagrass die-offs, of which wasting disease appeared to be a contributing factor, was the 1980s Florida Bay die-off, which significantly impacted *T*. *testudinum* beds [14, 41]. While numerous studies have been published that describe and characterize the interactions between *Zostera marina* (eelgrass) and *L*. *zosterae* [e.g. 7–9, 11, 13, 31–32, 36–39, 42–44], much less is known about *T*. *testudinum* and the *Labyrinthula* spp. which infect it. The ecological and commercial value of this species compels a greater understanding of its susceptibility to seagrass wasting disease.

Collectively, the goals of this study were to 1) generate a panel of immune biomarkers (POX, EXOC, PPO, and LYS activity) to provide an estimate of immune activity, 2) establish and test a qPCR assay that measures pathogen loading across a multitude of putatively seagrass-pathogenic *Labyrinthula* strains, and 3) investigate the role of common environmental stressors in regulating host immunity in *T*. *testudinum* while exploring the potential link between immune status and susceptibility to *Labyrinthula* sp. infection.

## Materials and methods

### Collection and maintenance of *Thalassia testudinum* and *Labyrinthula* cultures

*T*. *testudinum* shoots were collected near Horseshoe Bay (29°20’ N, 83°23’ W), off the Gulf Coast of Florida, USA, in May 2015. Permission to collect (permit SAL-15-1172-SR) was obtained through the Florida Fish and Wildlife Conservation Commission. Individuals were cleaned of epiphytes and transplanted, within 24 h of collection, to terra cotta pots filled with a 50:50 mixture of sediment collected from the sampling location, and Agra-Alive!^™^ (CaribSea Inc., Ft. Pierce, FL). Plants were maintained in aquaria at the University of North Florida greenhouse, as per methods previously described by Bishop et al. [6]. Briefly, plants were sustained at a salinity of 30 ppt, diel temperatures were between 25 and 27°C, and photosynthetically active radiation levels were maintained below 300 μmol m^-2^ s^-1^ [6]. At the time of their use in stress-response experiments, the average length and width of the longest blade (3^rd^ or 4^th^ rank) in each *T*. *testudinum* shoot was approximately 12.3 ± 2.8 cm and 0.35 ± 0.05 cm, respectively.

Cultures of *Labyrinthula* sp. “E” isolate 8b, a strain isolated from and known to readily infect *T*. *testudinum* [5–6, 33, 45] was used in infection trials throughout this study. The strain was maintained through growth on supplemented serum-seawater agar (SSA, described in [46]) plates with subsequent plate transfers occurring every 2–4 weeks. Briefly, SSA media was generated by combining 500 mL filtered and autoclaved seawater (at 25 ppt), 6 g agar, 0.5 g glucose, 0.05 g each of nutritional yeast and peptone, 1.5 mg germanium dioxide, 5 mL horse serum and 12.5 mL of a solution containing 1.25% each of streptomycin and penicillin (ex. 1.25 g each per 100 mL ddH_2_O).

### Experiment 1: Constitutive immune response

**Fig 1:**
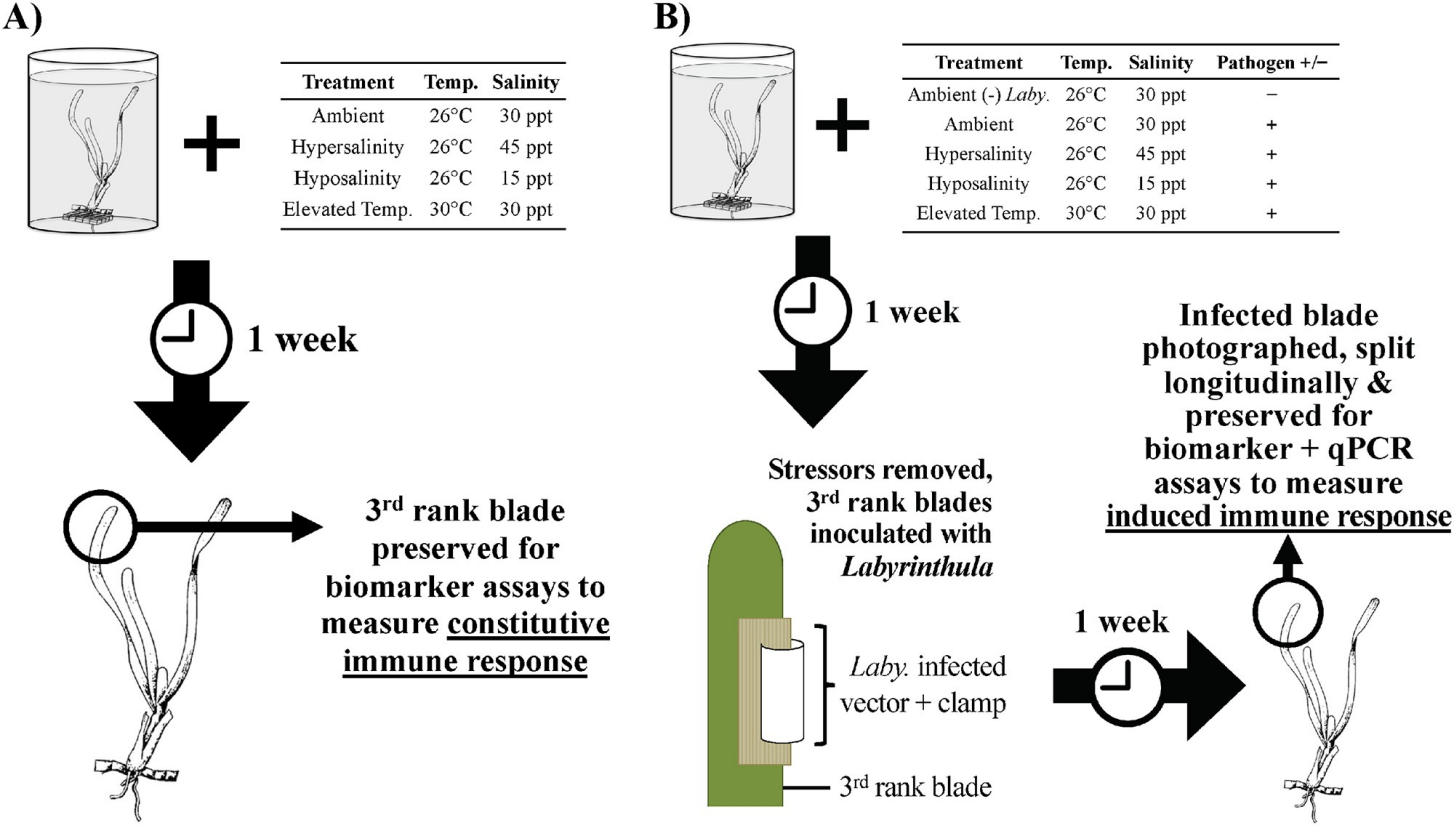
Workflow associated with seagrass (A) constitutive immunity assessment (Experiment 1) and (B) induced immunity assessment (Experiment 2) described in this study.

In order to determine how selected abiotic environmental stressors influence constitutive immunity in turtlegrass, whole *T*. *testudinum* shoots were exposed to these stressors under controlled laboratory settings. Briefly, individuals were randomly assigned to treatment groups (n = 5 per treatment) and subjected to either ambient temperature and salinity, elevated temperature, hypersalinity, or hyposalinity conditions in a microcosm environment for 7 d (Fig 1A). Microcosms were created using 3.8 L polyethylene terephthalate containers (Rubbermaid^©^, Winchester, VA, USA). Each container was equipped with a small water pump (Hydor^©^ Evolution Mini Pump, Sacramento, CA, USA) to promote circulation. Elevated temperature treatments were heated using submersible heaters (Hydor^©^, Sacramento, CA, USA). Instant Ocean Sea Salt (Instant Ocean^®^, Blacksburg, VA, USA) was used to create all desired salinities. The microcosms were assigned a position within the experimental setup using complete randomization to eliminate treatment effects due to location. Lighting was maintained at a photoperiod ratio of 12:12 h light:dark (~200 μm m^-2^ s^-1^), using full spectrum Power-FLO T5 HO bulbs (Hagen, West Yorkshire, UK). After 7 d under assigned conditions, tissue samples were extracted and processed, with no laboratory-based pathogen exposure. The third rank blade from each shoot was isolated and split longitudinally using a scalpel. Only one half was retained and stored at -80°C for use in immune biomarker assays to maintain consistency with sample handling in Experiment 2, where the other half of the blade was preserved for qPCR analyses.

#### Protein extraction from seagrass tissue

A section (~ 0.1 g, centrally located and adjacent to lesion, if present) of each *T*. *testudinum* blade (3^rd^ rank, split longitudinally) was combined with three steel beads in a microcentrifuge tube with 250 μL of extract buffer (0.2 M phosphate and 5 mM β-mercaptoethanol in HPLC-grade water, pH 7.8) and homogenized using a FastPrep^®^-24 Tissue Homogenizer (Irvine, CA, USA), at a speed of 4.0 m/s, repeated at 1 min intervals until the mixture resembled a fine paste. Samples were then centrifuged for 10 min at 17,000 x g and the resulting supernatant was transferred to a new tube. Following a second centrifugation for 15 min at 17,000 x g, the supernatant was transferred to a final tube and stored at -20°C for use in subsequent immune biomarker assays. Crude extract protein concentrations were obtained using a Pierce BCA Protein Assay Kit according to the published manufacturer’s protocol (Thermo Fisher Scientific^™^, Waltham, MA, USA). All subsequent immune assay activities were normalized by protein content and performed in clear, flat-bottom Fisherbrand^®^ 96-well plates (Thermo Fisher Scientific^™^, Waltham, MA, USA) unless noted otherwise.

#### POX activity

Crude extracts were diluted to a 10% solution by combining 5 μL sample extract and 45 μL of a 0.01 M phosphate buffer (pH 6.0). Forty-seven μL of 0.01 M phosphate buffer was added to each microplate well. Subsequently, 50 μL of 25 mM guaiacol (in 0.01 M phosphate buffer, pH 6.0) and 10 μL of the 10% extract solution were added. Finally, the reaction was initiated when 8 μL of 20 mM hydrogen peroxide (in 0.01 M phosphate buffer, pH 6.0) was added to each reaction well. Sample absorbance values were measured at 1 min intervals, for 20 min at 470 nm, using a Synergy^™^ HT Multi-Detection Microplate Reader (Winooski, VT, USA). POX activity for each sample was expressed as the change in absorbance at 470 nm min^-1^ μg^-1^ of total crude protein.

#### EXOC activity

Initially, 22.5 μL of assay buffer (100 mM sodium acetate, 0.1% SDS, 0.1% Triton X-100, 10 mM EDTA, 10 mM β-mercaptoethanol in HPLC-grade water, pH 5.0) was added to each microplate reaction well (black 96-well assay plate). Subsequently, 22.5 μL of crude sample extract was added to each well along with 45 μL of 0.5 mM 4-methylumbelliferyl *N*-acetyl-β-D-glucosaminide (Sigma Aldrich, Darmstadt, Germany) in sodium acetate buffer (pH 5.0). The contents of the wells were gently perturbed, ensuring no bubbles were present, after which the plate was covered in aluminum foil and incubated at 37°C for 30 min. Following incubation, fluorescence of the liberated product, 4-methylumbelliferone, was detected on a FLx800 Fluorescence Microplate Reader (BioTek Instruments, Winooski, VT, USA) with excitation and emission wavelengths of 360 and 460 nm, respectively. Sample fluorescence values were compared to a standard curve based on a dilution series of free methylumbelliferone (0–100 nM). EXOC activity was presented as nmol of 4-methylumbelliferone (MU) released per μg of total crude protein.

#### PPO activity

PPO activity for each sample was calculated using a modified version of the assay previously described in Gertzen and Escobar [47]. First, a 20 mM 3,4- Dihydroxy-L-phenylalanine (L-DOPA) substrate solution was made consisting of 100 mM sodium phosphate (pH 7.0) buffer with 0.15% SDS. To effectively solubilize the substrate, heat was gently applied as the solution was mixed. A very small volume (one medium-size pipette tip dipped in catalase powder per 10 mL solution) of catalase was added to the cooled solution. Two hundred μL of the substrate-containing solution was combined with 30 μL of crude sample extract in each microwell. The enzymatic conversion of L-DOPA to the oxidized dopachrome product was quantified by measuring the change in absorbance at 490 nm using a Synergy^™^ HT Multi-Detection Microplate Reader. PPO activity was presented as the change in absorbance at 490 nm min^-1^ μg^-1^ of total crude protein.

#### LYS activity

LYS or lysozyme-like activity was measured by monitoring the inhibition of *Micrococcus luteus* bacteria growth in the presence of crude sample extract, following a modified version of Couch et al. [48]. Briefly, freeze-dried *M*. *luteus* bacteria (MicroKwik Culture^®^, Carolina Biological Supply, Burlington, NC, USA) was rehydrated in media (provided in MicroKwik Culture^®^ kit) and subsequently diluted to 30% using a sodium phosphate buffer (10 mM phosphate buffer, pH 7.4). In one 96 well plate, 100 μL of the diluted *M*. *luteus* solution was combined with 25 μL crude sample extract (one well each, containing extract from each sample). A second plate served as a set of parallel blanks for each sample extract, where the *M*. *luteus* solution was replaced with 100 μL of the 10 mM phosphate buffer (pH 7.4). The absorbance of each sample/well was recorded at the start of the assay, and after approximately 24 h of incubation at room temperature, using a Synergy^™^ HT Multi-Detection Microplate Reader at 405 nm. LYS activity was quantified as the percent inhibition of *M*. *luteus* growth conferred by an individual’s crude protein extract (per μg of total crude protein), standardized by presenting the value as a function of the uninhibited growth of *M*. *luteus* in the absence of crude extract (averaged across 5+ plated replicate wells).

### Experiment 2: Induced immune response

To assess the induced immune response of *T*. *testudinum* due to abiotic stressors followed by laboratory-based pathogen exposure, an experiment similar to the first was conducted with minor differences. The goal of this experiment was to simulate a scenario where we attempt to isolate the impact of environmental stressors on the host seagrass species, and not the pathogen (by removing the stressor as the pathogen is introduced). First, after one week under assigned conditions, all individuals (n = 5 per treatment) were returned to ambient conditions (26°C, 30 ppt seawater) and inoculated with *Labyrinthula* sp. “E” isolate 8b [5] (e.g., a “+” *Labyrinthula* sp. treatment; or a “-” *Labyrinthula* sp. treatment, if a negative control), according to the methods outlined by Bishop et al. [6] (Fig 1B). Second, one-week post-inoculation, disease severity was quantified in post-treatment experimental samples using a modified version of the Wasting Index method [37]. Blades with necrotic lesions appearing on inoculated tissue were photographed digitally, and the proportion of diseased area measured using ImageJ software [49]. Third, post-imaging, tissue samples were processed by splitting the third rank blade longitudinally, with one half put into a 15 mL falcon tube and stored at -80°C for immune biomarker assays (as described above) and the other half preserved in Drierite^®^ (Sigma Aldrich, Darmstadt, Germany) for downstream qPCR analyses (see details below).

#### Detection of *Labyrinthula* spp. loading in seagrass tissue

In an effort to more reliably and precisely quantify the abundance of parasites in seagrass tissue, a novel qPCR-based approach was developed. Primers were designed to amplify a portion of the internal transcribed spacer (ITS) region of the ribosomal RNA gene complex for *Labyrinthula* spp. based on publicly available sequences from GenBank. Specifically, we targeted a 256 bp sequence of the ITS1 region with the novel primers LabPathITS1-3F (5’- CAA CTC AAT GAA TAT CTT GGT TTC C -3’) and LabPathITS1-3R (5’- CCG CTT ATT GAT ATG CTT AAA TTC -3’). Two *Labyrinthula* spp. isolates, *L*. sp. “E” isolate 8b and *L*. *zosterae* isolate 316b (Table 1), served as the primary strains utilized during optimization of the qPCR assay. Both isolates are robust, readily pathogenic to their respective hosts, and have been maintained on serum-seawater agar plates for 13 and 6 years, respectively. After the protocol was optimized, the method was used to detect pathogenic cells within experimentally inoculated *T*. *testudinum* and across 37 additional isolates of *Labyrinthula* spp. from culture (most originating from [5]). These isolates included both putatively pathogenic and non-pathogenic isolates collected opportunistically around the globe from a variety of temperate and tropical hosts (Table 1).

**Table 1:** *Labyrinthula* spp. isolates used to test the effectiveness of the qPCR assay. Twelve (12) putatively seagrass-pathogenic (“P”) and 27 seagrass non-pathogenic (“N”) *Labyrinthula* spp. isolates of various origins used to test the effectiveness of the qPCR assay. Isolate host/substrate code: ZM = *Zostera marina*; ZP = *Zostera pacifica*; PT = *Phylospadix torreyi*; PO = *Posidonia oceanica*; CN = *Cymodocea nodosa*; SI = *Syringodium filiforme*; HW = *Halodule wrightii*; TT = *Thalassia testudinum*; PS = *Phylospadix scouleri*; ML = detrital mangrove leaves; Px = *Phylospadix* sp.

Isolate ID Code	GenBank Access. No.	Isolate Host/ Substrate Code	Putative Functional Clade	Seagrass Bioregion Climate	Collection Site	qPCR results
107b	KU559383	ZM	P	Temperate	USA, NY, Long Island, Fisher Island	+
113b	KU559384	ZP	P	Temperate	USA, CA, Refugio Beach	+
142b	KU559395	ZP	P	Temperate	USA, CA, Anacapa Island	+
140b	KU559419	PT	P	Temperate	USA, CA, Goleta, Coal Oil Point	+
302b9	KU559426 KU559431 KU559431	PO	P	Temperate	Spain, Alicante Bay	+
287b	KU559431	CN	P	Temperate	Spain, Cabrera Island, Port Cabrera	+
180b3	KU559437 KU559441 KU559441	SI	P	Tropical	Australia, Brisbane, N. Stradbroke Island	+
74b	KU559441	HW	P	Tropical	USA, FL, Key West	+
160b1	KU559451 KU559457 KU559457	TT	P	Tropical	Panama, Playa Longosta	+
8b	KU559457	TT	P	Tropical	USA, FL, Perdido Key	+
316b	KU559372	ZM	P	Temperate	USA, OR, Newport	+
347b	(none*)	ZM	P	Temperate	USA, VA	+
30b	KU559462	PS	N	Temperate	USA, WA, San Juan Island, Cattle Point	−
282b	KU559470	ML	N	Tropical	Belize, Twin Cay	−
229b	KU559473	TT	N	Tropical	Bahamas, Exuma Cay, Big Point	−
109b	KU559480	ZM	N	Temperate	Portugal, Ria Formosa	−
75b	KU559488	HW	N	Tropical	USA, FL, Key West, 2	−
84b	KU559491	HW	N	Tropical	USA, FL, Perdido Key	−
239b	KU559500	ZM	N	Temperate	USA, WA, Sucia Island, Shallow Bay	−
243b	KU559501	ZM	N	Temperate	USA, WA, Sucia Island, Shallow Bay	−
250b	KU559502	Px	N	Temperate	USA, CA, Cayucos	−
255b	KU559503	ZM	N	Temperate	USA, CA, Morro Bay	−
267b	KU559504	Px	N	Temperate	USA, CA, Cayucos	−
271b	KU559506	ZM	N	Temperate	USA, WA, Shaw Island, Picnic Cove	−
50w	KU559516	ZM	N	Temperate	USA, WA, Shaw Island, Picnic Cove	−
51b	KU559517	ZM	N	Temperate	USA, CA, San Diego Bay, Shelter Island	−
214b	KU559524	ZM	N	Temperate	USA, VA, Chesapeake Bay, Cape Charles	−
254b	KU559530	ZM	N	Temperate	USA, CA, Morro Bay	−
237b	KU559541	ZM	N	Temperate	USA, WA, Lopez Island, Shoal Bay	−
295b	KU559545	ML	N	Tropical	Belize, Twin Cay	−
345b	(none*)	ZM	N	Temperate	USA, VA	−
66b	KU559487	TT	N	Tropical	USA, FL, Marquesas	p
77b	KU559489	HW	N	Tropical	USA, FL, Marquesas	p
48b	KU559515	PS	N	Temperate	USA, CA, La Jolla Shores	p
52b	KU559518	Px	N	Temperate	USA, CA, La Jolla Shores	p
279b	KU559486	TT	N	Tropical	Belize, Twin Cay	+
98b	KU559522	ZM	N	Temperate	USA, WA, San Juan Island, False Bay	+
161b3	KU559529	ML	N	Tropical	Panama, Playa Longosta	+
284b	KU559546	ML	N	Tropical	Belize, Twin Cay	+

*Putative validation by morphology and substrate (D.L. Martin, pers. obsv.). Seagrass bioregions and their climate are as defined by Short et al [50]. Results of the new primer pair for particular isolates are summarized as positive (+), negative (−), and partial (p) ‘hits’ when using qPCR (see text for qualifying remarks on “p” and “+” hits for N-clade types).

#### DNA extraction

Longitudinal half-leaf sections were first pulverized using three steel beads in a microcentrifuge tube with a FastPrep^®^-24 Tissue Homogenizer (MP Biomedicals, Solon, OH, USA), at a speed of 4.0 m s^-1^, repeated at 1 min intervals until the mixture resembled a fine powder. Next, DNA was extracted from a ~5.0 mg subsample of the pulverized tissue using an Invisorb^®^ Spin Tissue Mini Kit (Stratec Molecular, Berlin, Germany), according to the manufacturer’s protocol. The resulting eluent was purified using a OneStep^™^ PCR Inhibitor Removal Kit (Zymo Research, Irvine, CA, USA). Prior to use in qPCR assays, the concentration of each DNA sample was measured using an Epoch Microplate Spectrophotometer (BioTek Instruments, Winooski, VT, USA) and adjusted to reflect a standardized concentration of 10 ng μL^-1^. When adding 2 μL of template DNA to later qPCR reactions, this resulted in a final concentration of 1 ng μL^-1^ DNA template per total reaction volume (20 μL).

#### Cell counts/standard curve

Cell counts were performed on *Labyrinthula* sp. “E” isolate 8b cells which had been transferred from the standard, long-term serum-seawater agar media to a liquid medium (formulated as stated above minus the agar). The cultures were grown for 24 h at room temperature, gently scraped off the surface of culture plates, and resuspended in 1 mL of liquid culture containing 2% Tween 80^®^ (Sigma Aldrich, Darmstadt, Germany) for every five growth plates, followed by a 15 s vortex to help disrupt cell clumps. If large clumps remained, a new batch of plates was used. Then, 10 μL of cell culture was loaded into a Neubauer-improved hemocytometer grid and four of the nine grid cells were counted at 400x magnification. This process was repeated with a total of 28 aliquots to generate a 1X standard solution of ~1,290 cells μL^-1^. Genomic DNA was extracted from these aliquots as described above. This stock solution was used to generate aliquots of DNA corresponding to *Labyrinthula* sp. cell concentrations of 320, 81, 5.0, 0.31, 0.02, and 0.0012 cells μL^-1^, which was used for the standard curve in all qPCR runs. Finally, extraction efficiency, which assesses whether PCR amplification is consistent across a broad range of template concentrations, was calculated for the qPCR procedure using the linear regression slope of the standard curve, in the following equation: efficiency = 10^(-1/slope)^ −1 [39].

#### qPCR protocol

The qPCR reaction consisted of the following final concentrations: 1 ng μL^-1^ of DNA template, 0.025 μM of each primer, 2.7 ng μL^-1^ of BSA, 1X of iTaq SYBR Green Supermix (Bio-Rad Laboratories, Hercules, CA, USA), and UltraPure^™^ DNase/RNase-Free water (Invitrogen^™^ of Thermo Fisher Scientific^™^, Waltham, MA, USA) brought to a final volume of 20 μL. Reactions were run on a CFX Connect Real-Time PCR Detection System (Bio-Rad Laboratories, Hercules, CA, USA) using the following protocol: 95°C for five min, followed by 45 rounds of a two-step thermocycling protocol (95°C for 30s, 63°C for 60s). At the end of each cycle, fluorescent signals were read and recorded. Finally, a melting curve step was performed (65–95°C, at 0.5°C increments) to help verify the identity of a specific amplicon. All samples were run in triplicate and a negative control (well containing PCR reagents + water in lieu of template DNA) was run on each plate to ensure that contamination and/or false positives were not occurring.

#### Correlating individual immune responses and pathogen loading

In order to address the potential role of individual variation in the host response to abiotic stressors and/or susceptibility to SWD, the association between immune biomarker (POX, EXOC, PPO, and LYS) activity and pathogen loading was assessed by pooling together all individuals from the second stress-response experiment, regardless of treatment. This data was used to correlate activity levels of each biomarker with pathogen load, and to create a heat map to allow for the simultaneous visualization of the five parameters measured (POX, EXOC, PPO, and LYS activity, and pathogen load) for each individual. This was achieved using the expression visualization tool of Heatmapper [51], scaling and shading values by the column z-score which corresponded with the magnitude of that variable (blue, white, and red representing Z-scores of -4, 0, and 4, respectively). Individuals were reordered from lowest to highest pathogen load values to view trends in the immune markers paired by individual.

### Statistical analyses

All statistical tests were performed with 95% confidence intervals (α = 0.05) on data that were normally distributed according to Kolmogorov-Smirnov and Sharpiro-Wilk normality tests, unless stated otherwise. All statistical tests were computed using IBM SPSS^®^ Statistics 25 software (International Business Machines Corp., Armonk, NY, USA) unless stated otherwise.

For both experiments, one-way ANOVAs were conducted to test for differences among treatment groups on measurements representing either POX, EXOC, PPO, and LYS activities, and pathogen load (when applicable). In two instances (Experiment 1 EXOC activity and Experiment 2 EXOC activity), the assumption of homogeneity of variances was violated; however, it was noted that these ANOVA results were non-significant and, because the nonparametric alternative (i.e. the Kruskal-Wallis test) is less likely to find significance than a classic ANOVA [52], it was assumed that the parametric ANOVA results held valid. Following one-way ANOVA tests, individual treatment groups were compared pairwise by multiple comparisons LSD post hoc tests (α < 0.05). All immune biomarker raw values were log transformed to meet the assumptions of normality. In the second experiment, LYS values could not be transformed to meet the assumptions of normality, but ANOVA was considered robust enough to handle this violation (as demonstrated in [53]).

When comparing immune biomarker activity with pathogen load using the pooled values across all treatments from Experiment 2, POX, EXOC, and PPO values were transformed using natural log to meet the assumptions of normality, and Pearson product-moment correlation coefficients, along with coefficient of determination (R^2^) and p-values, were calculated using these transformed variables. LYS values could not be transformed to meet the assumptions of normality. Thus, pathogen loading and LYS activity were ranked and the Spearman’s rho nonparametric equivalent was utilized to assess the strength of their linear relationship.

Pathogen growth in terms of load per mg dry weight of leaf, quantified via qPCR, was compared to Wasting Index [37] estimates of disease severity using regression analyses. Akaike’s information criterion adjusted for small sample size (AICc) was used to determine the best model using Prism8.0.1 (GraphPad Software Inc., San Diego, CA, USA). Initial model choice included linear, segmental linear, logistic and Gompertz, as we hypothesized that: 1) lesions appear after some threshold level of parasite abundance, 2) the relationship between lesion area and cell count will decrease at larger lesion sizes due to higher parasite abundance at lesion margins [43], and 3) proliferation of cells as a function of lesion size would be the result of complex host-pathogen interactions likely approaching a sigmoidal response (when considering lesion size as a rough proxy for time)–as is common for microbe growth in culture media. A logistic model was used incorporating the following equation: Y = Y_M_*Y_0_/((Y_M_-Y_0_)*exp(-k*x) where Y_0_ represents the starting population, Y_M_ represents the maximal population and k is the rate constant.

## Results

### Experiment 1: Constitutive immune response experiment

**Fig 2:**
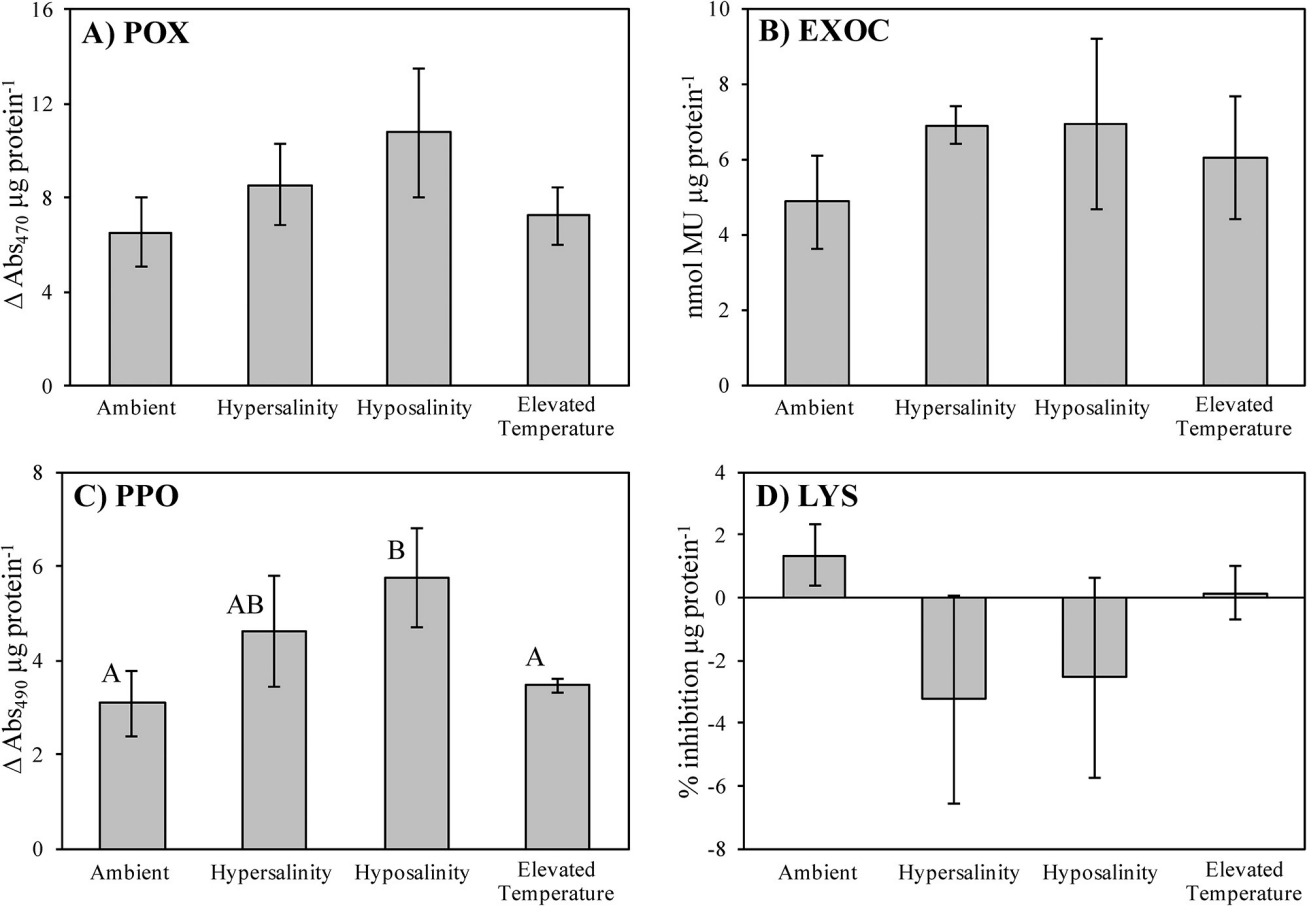
Immune responses in *T*. *testudinum* following abiotic stress. Immune biomarker activity levels (A-D: peroxidase, exochitinase, polyphenol oxidase, and lysozyme, respectively) of *T*. *testudinum* individuals from Experiment 1 are shown under various treatments (control/ambient, hypersalinity, hyposalinity, or elevated temperature). Error bars reflect ±1 SEM. Letters represent significant differences in mean activity levels among treatment groups as determined by ANOVA (see S1 Table for details), followed by multiple comparisons LSD post hoc tests (two-tailed, α = 0.05; see S2 Table for details). Sample size n = 5, with the following exceptions: hypersalinity and elevated temperature groups, n = 4 each.

Immune activities yielded differential responses with a notable level of variability both within and among treatment groups. POX, EXOC, and LYS activities failed to show any significant differences as a function of abiotic stress treatment (Fig 2A, 2B and 2D; see S1 and S2 Tables for details). However, PPO activity differed significantly between ambient and hyposaline (p = 0.02) and elevated temperature and hyposaline (p = 0.05) treatments (Fig 2C; S1 and S2 Tables).

### Experiment 2: Effectiveness of the qPCR procedure

**Fig 3:**
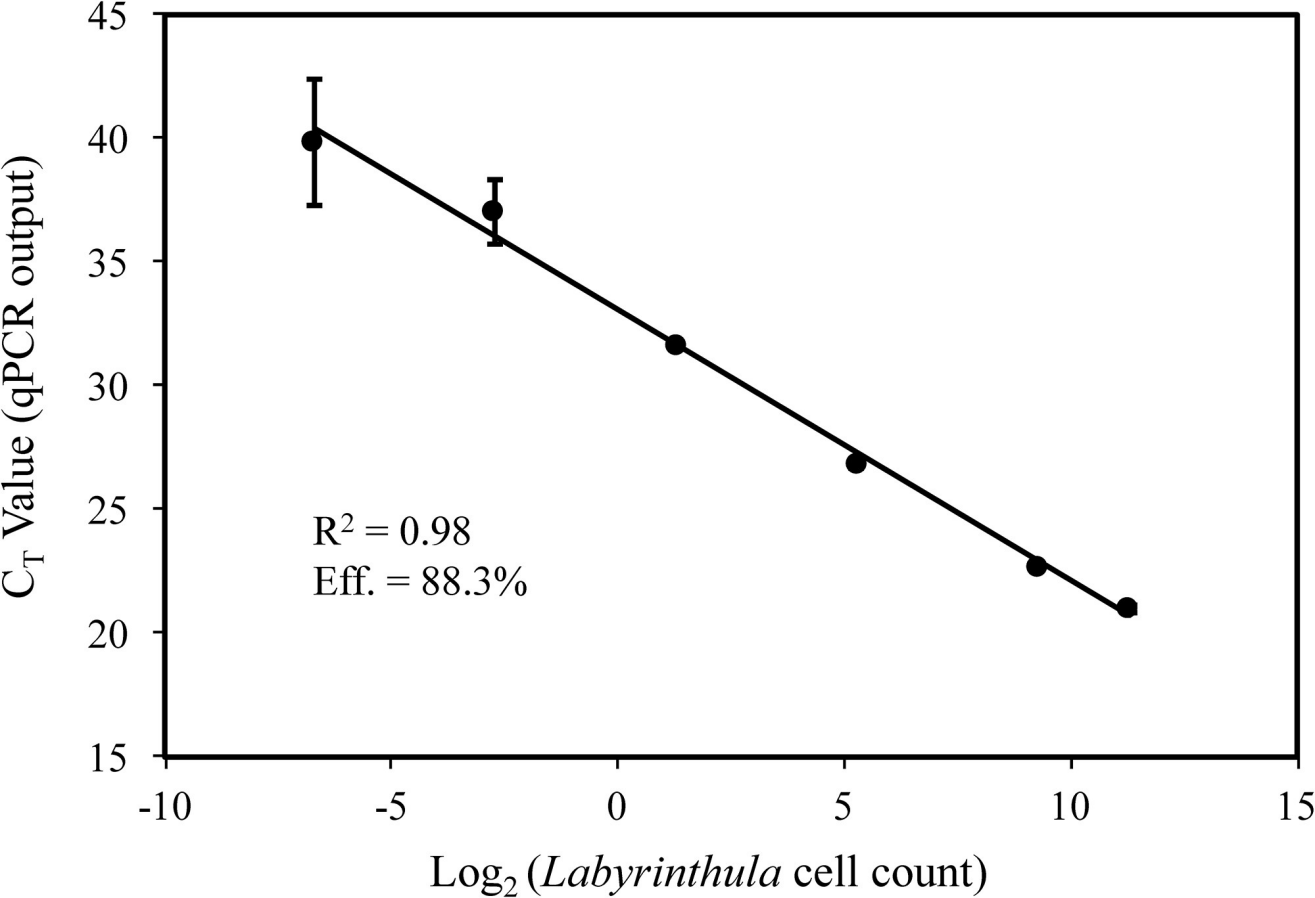
qPCR extraction efficiency. The number of *Labyrinthula* sp. “E” isolate 8b cells μL^-1^ (log_2_) across a cell dilution series is plotted against threshold (C_T_) values to calculate extraction efficiency. Each sample was run in triplicate. Error bars represent ±1 SEM.

The dilution series created with known cell counts of the seagrass-pathogenic *Labyrinthula* sp. “E” isolate 8b was utilized to generate a standard curve to assess the efficiency and precision of the qPCR assay presented herein by analyzing whether the assay results were proportional to the known cell number from which the DNA template was extracted. The resulting standard curve generated an R^2^ value of 0.98 and the extraction efficiency was 88.3% (Fig 3). Additionally, the qPCR assay reliably amplified the highest dilution within the series, indicating that the assay was capable of detecting pathogen DNA equivalent to approximately 0.0012 *Labyrinthula* cells μL^-1^ (or 0.024 cells per 20 μL qPCR reaction).

Our qPCR assay was tested on 39 cultured strains within the genus, including 12 putatively seagrass-pathogenic and 27 putatively seagrass-non-pathogenic *Labyrinthula*. The qPCR assay reliably amplified 100% of the putatively pathogenic stains (Table 1). Of the 27 putatively non-pathogenic strains, 70% never amplified, 15% reliably amplified, and 15% weakly amplified (i.e., the latter amplified at a late cycle and only in one of the two tests run on that strain).

**Fig 4:**
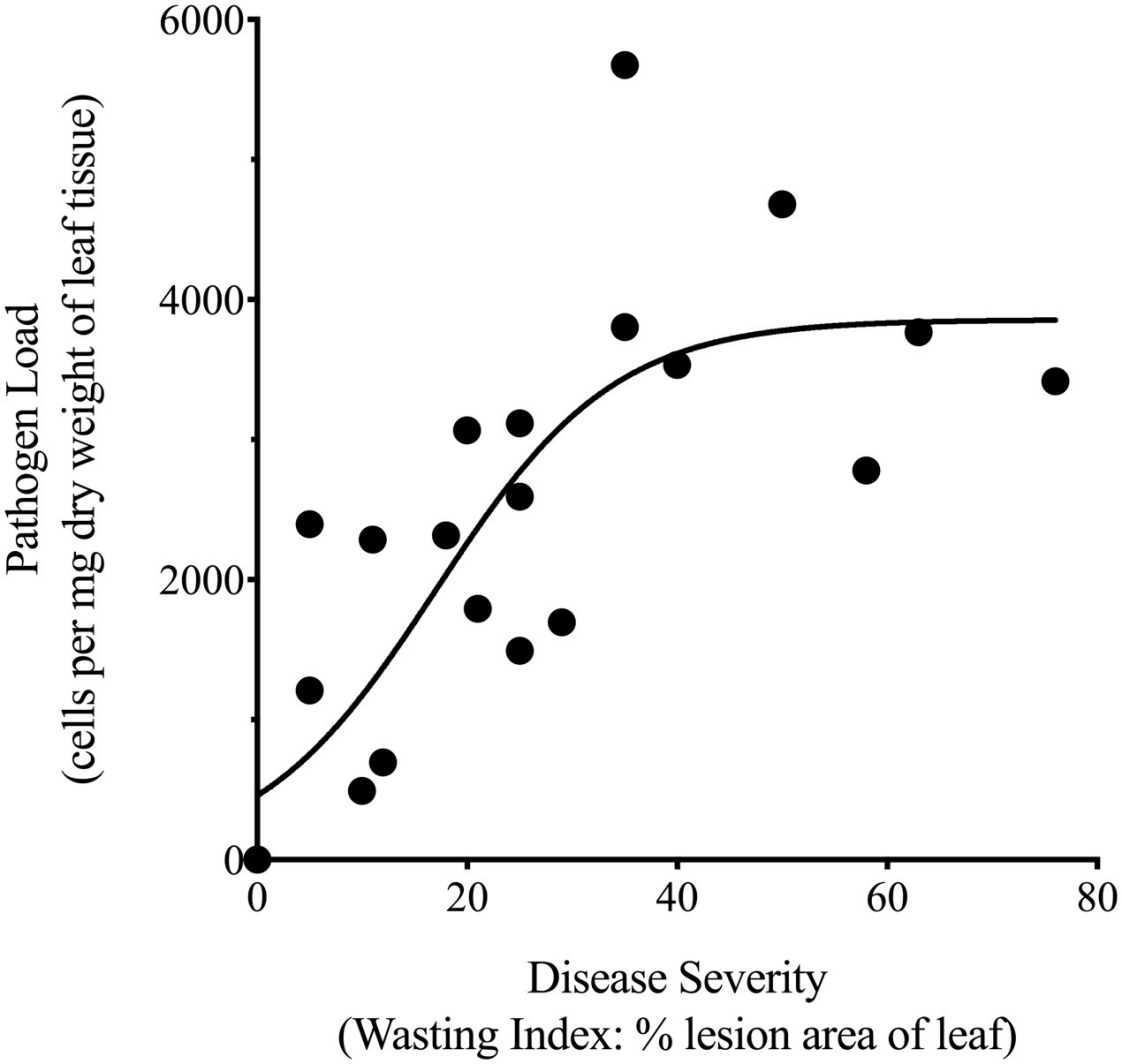
Relationship between *Labyrinthula* sp. “E” isolate 8b cell count values obtained through qPCR and percent leaf area coverage of ‘induced lesions’ as determined through Wasting Index methods. AICc provided the final logistic model choice (R^2^ = 0.68). See S3 Table for raw data.

The novel qPCR assay presented here successfully quantified putative seagrass-pathogenic *Labyrinthula* spp. abundance in host tissue, as evidenced by the results of Experiment 2. Additionally, the same melt-peak was observed in the experimental samples and the extraction from cultured *Labyrinthula*. sp. “E” isolate 8b (i.e. both with sharp peaks at 76°C). When we compared the qPCR assay with the historical Wasting Index (WI) method, we found a significant positive relationship between estimates of seagrass-pathogenic *Labyrinthula* spp. cell counts and percent leaf coverage of induced lesions (e.g., linear regression; p < 0.01, R^2^ = 0.54, n = 23; raw data in S3 Table). However, the AICc analysis indicates a logistic model provides the better estimate (R^2^ = 0.68), suggesting pathogen load does not scale linearly across lesion sizes as cell counts decline relative to larger lesion areas (i.e., > ~1/3 of leaf area; Fig 4).

### Experiment 2: Induced immunity of seagrass exposed to abiotic stressors followed by pathogen exposure

**Fig 5:**
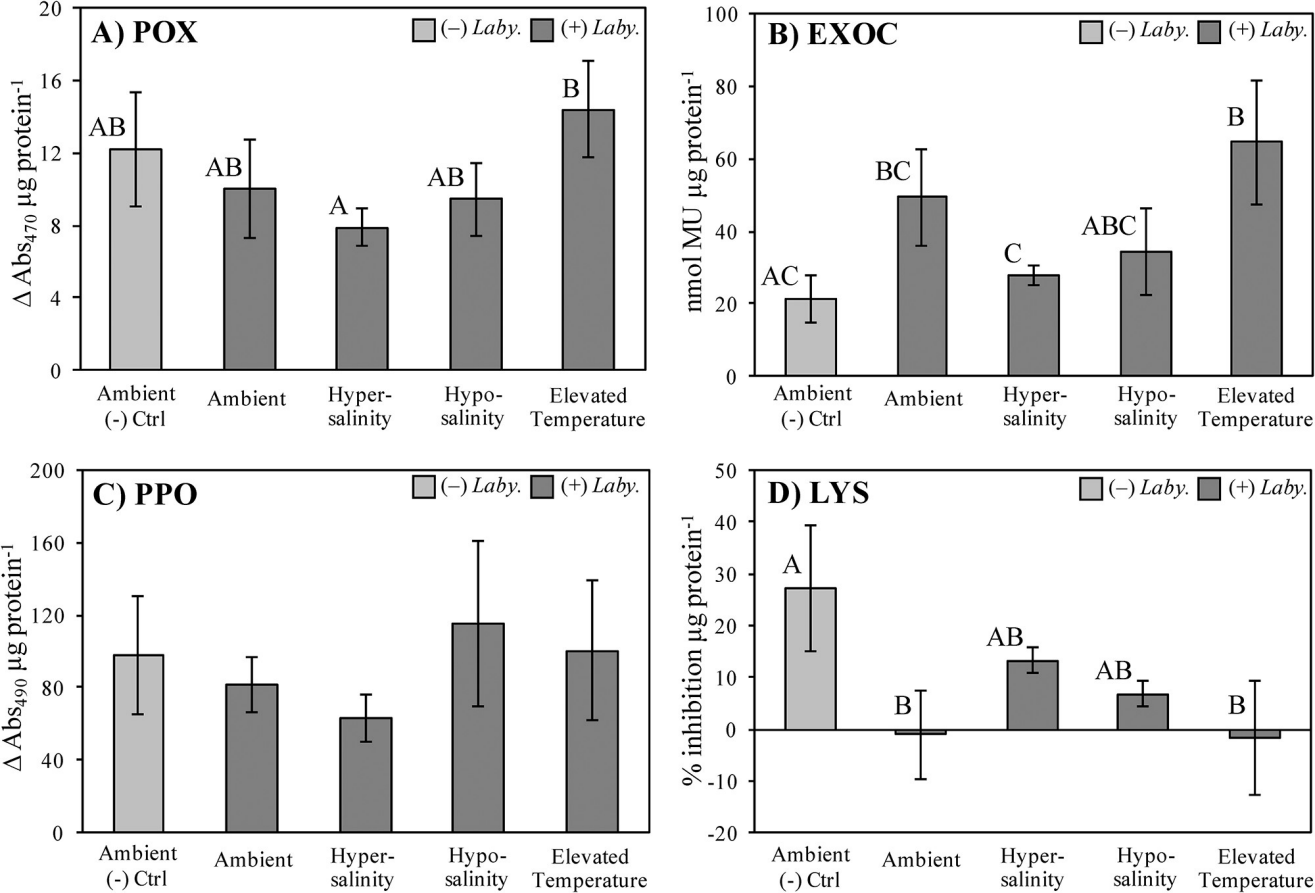
Immune response in *T*. *testudinum* following abiotic stress and *Labyrinthula* sp. infection. Immune biomarker activity levels (parts A-D: peroxidase, exochitinase, polyphenol oxidase, and lysozyme, respectively) of *T*. *testudinum* individuals from Experiment 2 are shown under various treatments [control/ambient (“-”) *Laby*.; ambient (“+”) *Laby*.; hypersalinity (“+”) *Laby*.; hyposalinity (“+”) *Laby*.; elevated temperature (“+”) *Laby*.]. Error bars reflect ±1 SEM. Different letters represent significant differences in mean activity levels among treatment groups as determined by ANOVA (see S4 Table for details), followed by multiple comparisons LSD post hoc tests (two- tailed, α = 0.05; see S5 Table for details). Sample size n = 5, with one exception: EXOC, hyposalinity treatment, n = 4.

Both POX and EXOC showed significantly lower activities under hypersalinity (“+” *Labyrinthula* sp. treatment) conditions when compared to the elevated temperature (“+” *Labyrinthula* sp.) treatment (POX activity p = 0.05; EXOC activity p = 0.04; Fig 5A and 5B; see S4 and S5 Tables for details), while PPO activity failed to show any significant differences among treatment groups (Fig 5C; S4 and S5 Tables). In addition, EXOC activities differed significantly between ambient control conditions (“-” *Labyrinthula* sp.) and elevated temperature (“+” *Labyrinthula* sp.) (p = 0.007; Fig 5B; S4 and S5 Tables). Significantly different LYS activities were also observed between ambient control conditions (“-” *Labyrinthula* sp.) and elevated temperature (“+” *Labyrinthula* sp.) (p = 0.03; Fig 5D; S4 and S5 Tables) as well as ambient control (“-” *Labyrinthula* sp.) and ambient (“+” *Labyrinthula* sp.) conditions (p = 0.03; Fig 5D; S4 and S5 Tables).

**Fig 6:**
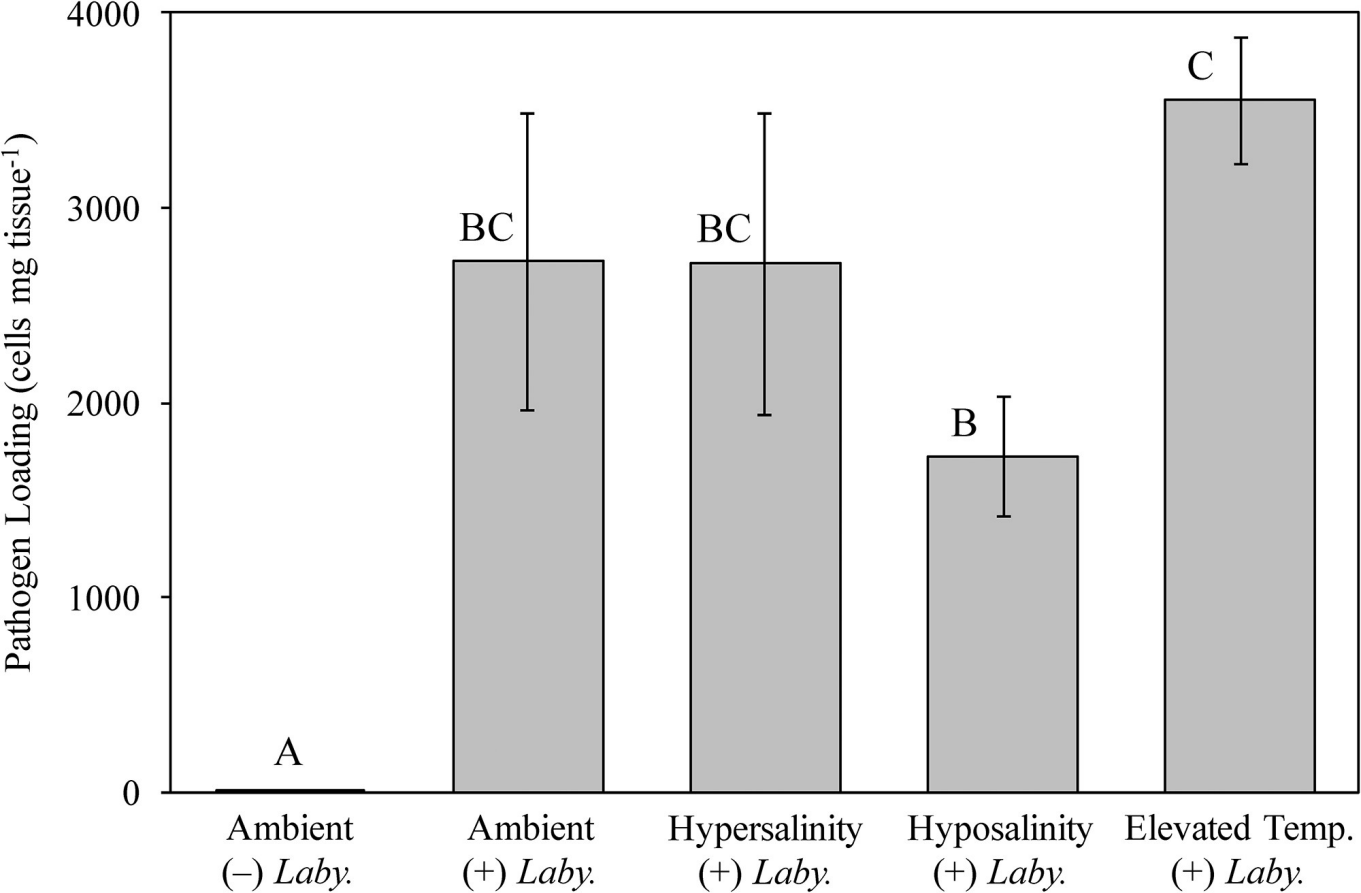
Infection responses in *T*. *testudinum*. Results of qPCR assays measuring pathogen (number of *Labyrinthula* sp. “E” isolate 8b cells mg tissue ^-1^) in *T*. *testudinum* individuals from Experiment 2, under control [no *Labyrinthula* sp., or (“-”) *Laby*.] and pathogen-exposed [ambient (“+”) *Laby*.; hypersalinity (“+”) *Laby*.; hyposalinity (“+”) *Laby*.; elevated temperature (“+”) Laby.] conditions. Error bars reflect ±1 SEM. Different letters represent significant differences in mean activity levels among treatment groups as determined by ANOVA, followed by multiple comparisons LSD post hoc tests (two- tailed, α = 0.05). Sample size n = 5, with one exception: Ambient (“-”) *Laby*. treatment, n = 4.

Our qPCR-based method detected ‘background’ abundances of *Labyrinthula* spp. cells in individuals belonging to the negative control treatment (Experiment 2: ambient conditions, “-” *Labyrinthula*) that were several orders of magnitude lower than found in all other treatment groups (p < 0.01 in all cases, Fig 6; see S6 Table for details), suggesting pathogen exposure under our laboratory conditions resulted in much higher pathogen loading than was found naturally in the shoots we used. Additionally, there was a significant difference between individuals belonging to the hyposalinity (“+” *Labyrinthula* sp.) and the elevated temperature (“+” *Labyrinthula* sp.) treatment groups (Fig 6; see S6 Table for details). However, there were no detectable differences between any of the abiotic stress treatments and the ambient control exposed to *Labyrinthula* sp. “E” isolate 8b (Fig 6; S6 Table). Furthermore, a subsequent ANOVA in which the significantly different negative control group (ambient conditions, “-” *Labyrinthula* sp., see S7 Table for details) was removed confirmed high levels of intra-treatment variability. The sum of square values within groups vastly exceeded that which measured between group variation (SS = 23041542.33 verses 8421011.47, respectively) among individuals who were introduced to the pathogen (see S8 Table for details).

**Fig 7:**
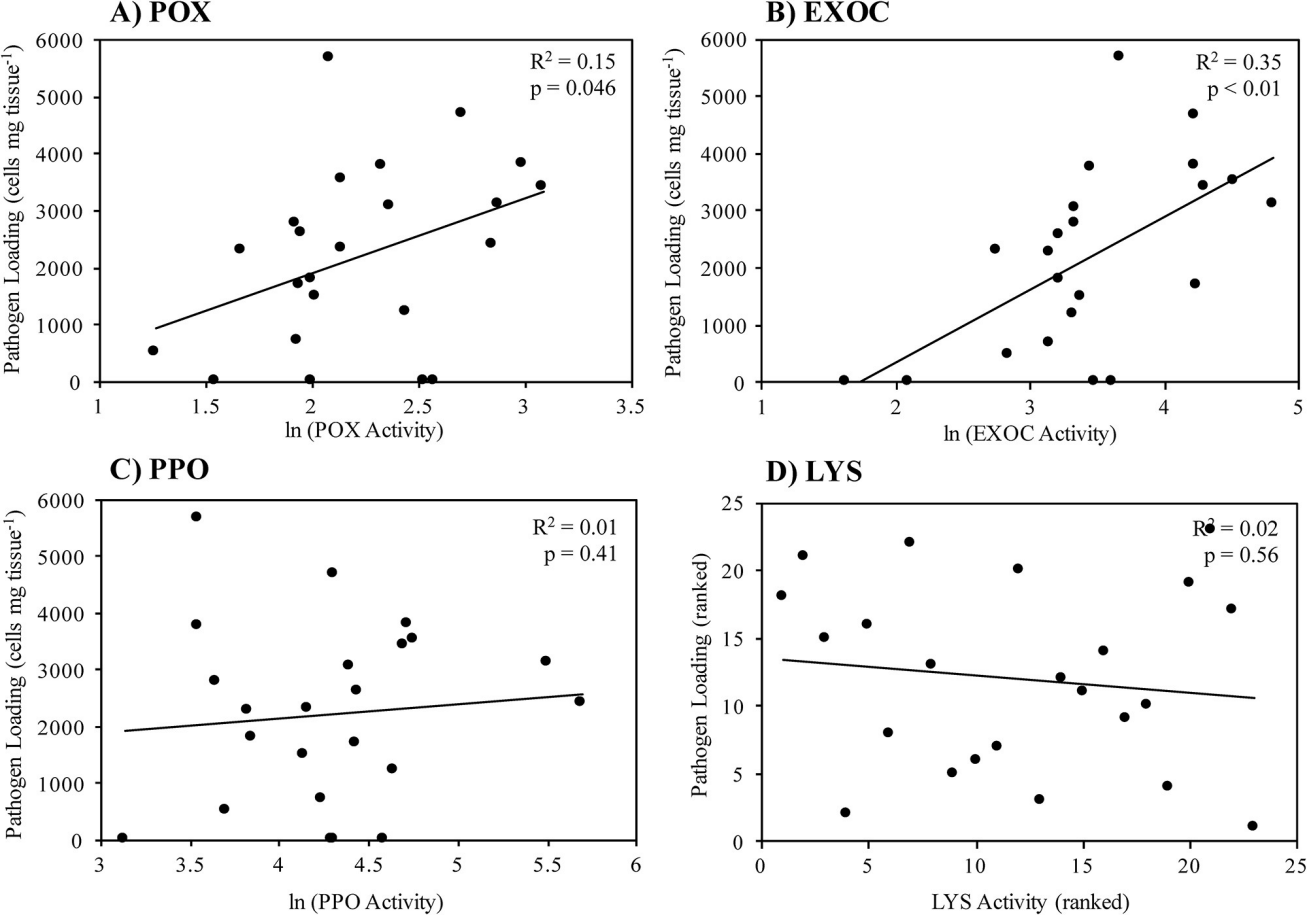
Results of correlation analyses between pathogen load (number of *Labyrinthula* sp. “E” isolate 8b cells mg tissue^-1^) and immune biomarker activity (parts A-D: Peroxidase, exochitinase, polyphenol oxidase, and lysozyme, respectively) in *T*. *testudinum* individuals from Experiment 2. Significance (p-values) are based on Pearson product-moment correlation coefficients (A-C) or Spearman’s rho correlation coefficients (D). Sample size in parts A, C and D = 24; sample size in part B = 23.

To address this variation, we tested the strength of relationships between each immune biomarker and *Labyrinthula* spp. cell count as a function of each individual. Correlative analyses revealed that there were significant positive relationships between individual pathogen load and POX activity (R^2^ = 0.15; p = 0.046; Fig 7A) and EXOC activity (R^2^ = 0.35; p < 0.01; Fig 7B). The relationships between pathogen loading and PPO activity or LYS activity were not significant (Fig 7C and 7D).

**Fig 8:**
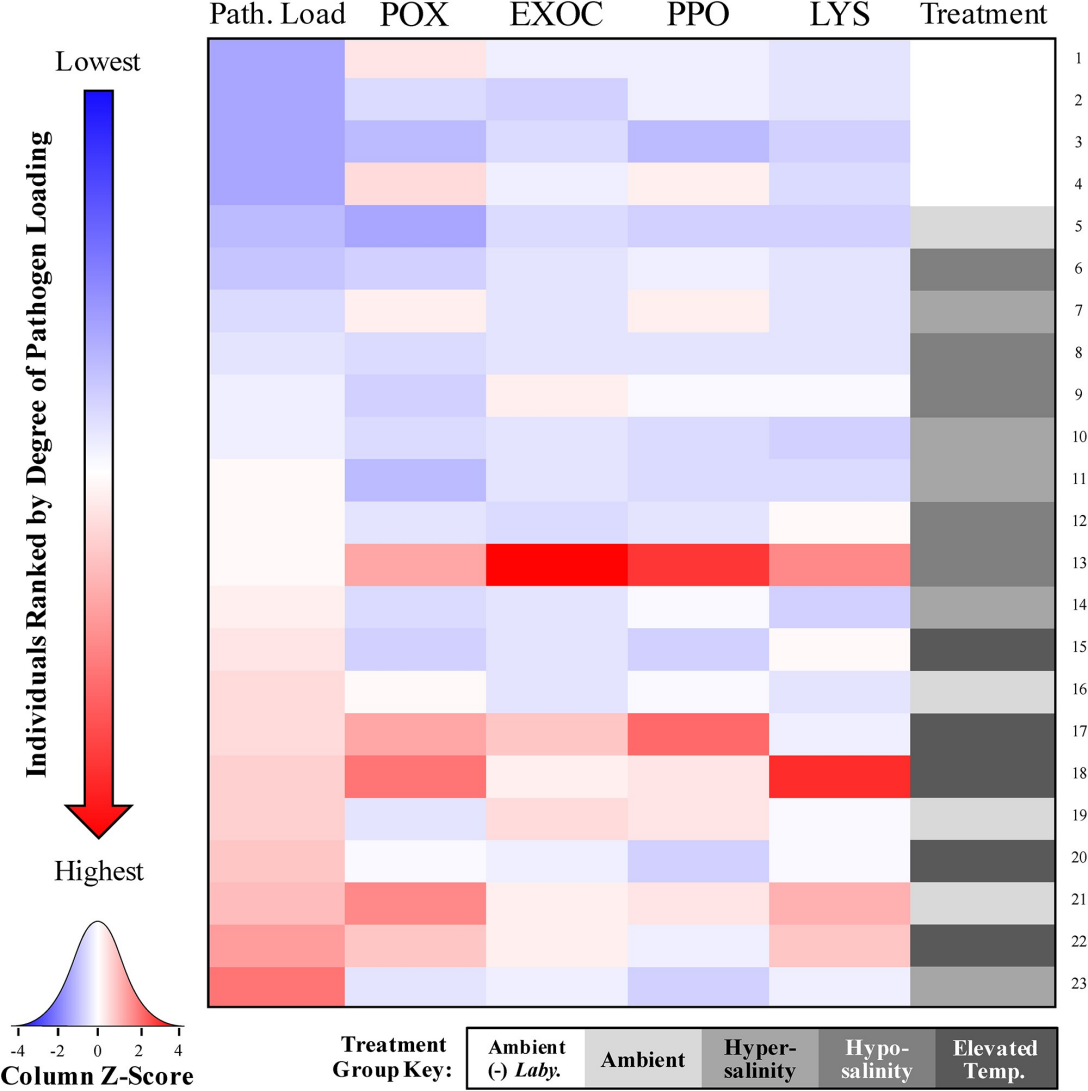
Association of immune responses and pathogen loading in *T*. *testudinum*. The heat map displays relative values of the five parameters measured (POX, EXOC, PPO, and LYS activity, and pathogen load) in *T*. *testudinum* individuals in Experiment 2 [subjected to control/ambient (“–”) *Labyrinthula* sp., ambient (“+”) *Labyrinthula* sp., hypersalinity (“+”) *Labyrinthula* sp., hyposalinity (“+”) *Labyrinthula* sp., or elevated temperature (“+”) *Labyrinthula* sp., shaded white, gray, light blue, blue, and dark blue, respectively]. Each column represents the relative pathogen load (*Labyrinthula* sp. “E” isolate 8b) or activity at each of the four biomarkers. In addition, each row represents one individual from the second stress-response experiment (in ascending order of pathogen loading, from top to bottom). The shade assigned to each individual at a given parameter reflects the Z-score of proportional activity at that parameter scaled by column, with blue, white, and red representing Z-scores of -4, 0, and 4, respectively.

The heat map (Fig 8) demonstrates the high variation among individuals in the second experiment, with individual “23” possessing the highest relative *Labyrinthula* spp. cell count, but relatively low levels of each of the four biomarkers (as indicated by the blue shading; Fig 8). Conversely, the plants with the 2^nd^ and 3^rd^ highest relative *Labyrinthula* spp. cell counts (“22” and “21”) have relatively high levels of the four biomarkers (7 of 8 relevant cells are shaded red; Fig 8). Further, there are few clear patterns of treatment group associated with the ranked order from lowest to highest pathogen loading, aside from the observation that the four plants with the lowest relative *Labyrinthula* spp. cell counts belong to the treatment where *Labyrinthula* sp. “E” was not added, and that all 5 of the individuals assigned to the elevated temperature treatment fall within the top 9 most-infected individuals (“15” through “23”; Fig 8). Individual “13” stands out as having very high biomarker activity, but falls in the middle of the z-score distribution in terms of pathogen loading (indicated by white shading; Fig 8), and the other replicates belonging to the same treatment (hyposalinity) do not share similar trends in biomarker activity (Fig 8).

## Discussion

Elucidating the relationship between aquatic species and their infectious diseases is pertinent in marine ecology because infection outbreaks have the potential to drastically alter ecosystem functionality (reviewed in [1, 3, 54]). Thus, the methods validated through this study provide a framework to better understand the complex dynamics of SWD. Herein we show that the four immune biomarkers assessed were constitutively active in turtlegrass individuals, but specific activities were largely unaffected by the chosen abiotic conditions provided. In addition, the qPCR methodology presented has the capability of successfully quantifying *Labyrinthula* spp. cells from both cultures and seagrass tissues across a broad range of predominately seagrass-pathogenic strains, with high sensitivity. Finally, the positive correlations between pathogen loading and peroxidase and exochitinase demonstrate the potential utility of these biomarkers in future applications. However, the results of this study highlight the complexity of plant immune responses.

### Biomarkers are constitutively active and variable among individuals, regardless of treatment

The results of the biomarker assays testing innate stress responses of the host in the presence of abiotic stressors indicate that POX, EXOC, and PPO were constitutively active in *T*. *testudinum* experimental individuals. We are using the term constitutive in this study to characterize the presence of enzyme activity in seagrass tissues that were never intentionally infected with *Labyrinthula* sp. “E” isolate 8b. Thus, it should be noted that this study did not use naïve hosts, so there could have been “trace” amounts of pathogen associated with the negative controls. Given that the trace amounts were orders of magnitude lower than intentionally infected individuals (Fig 6, S3 Table), it seems unlikely that such background levels “induced” the observed constitutive values reported.

It was hypothesized that constitutive expression of the chosen biomarkers in seagrass tissue may be compromised or suppressed in the presence of selected abiotic stressors. However, the results of Experiment 1 show that POX, EXOC, and LYS activity levels were not significantly affected by the stressors imposed (hypersalinity, hyposalinity, and elevated temperature) when compared to control groups. Thus, turtlegrass appears fairly resilient to the short-term duration (7 d) of the abiotic stressors we applied, at least with respect to these biomarkers. The only biomarker whose expression varied significantly among treatments was PPO (Fig 2C). Individuals exposed to hyposaline conditions had significantly higher PPO activities compared to those in either ambient or elevated temperature treatments.

Osmotic stress has been previously shown to increase PPO activity in a diverse array of plant taxa [55–56]. As hypo- or hypersaline conditions can induce oxidative stress in seagrasses [45], it is possible that increased PPO activity reflects enhanced anti-oxidant capacity by reducing the local concentrations of oxygen through the reduction of O_2_ to H_2_O [57]. Thus, at least with regard to the immune markers used in this study, constitutive immunity does not appear compromised by acute abiotic stress and might therefore continue to play a role as an active basal defense in preventing SWD development.

Generally speaking, there was a large amount of variability in biomarker activity across and within treatment groups. In future studies, this variability may be reduced, in part, by adopting a more systematic approach to how tissue was chosen from the blade for isolation; we recommend the entire sample be homogenized and then subsampled for a standardized amount to be used in extraction and downstream analyses to minimize bias that may be introduced by sampling a sub-region of tissue from within one seagrass blade.

### Pathogen detection via the qPCR assay

Our results indicate that the qPCR assay presented here is likely to detect a broad range of seagrass-pathogenic *Labyrinthula* spp. and/or strains (sensu [5]) from both temperate and tropical regions. The ability for this assay to show a positive selection bias of pathogenic relative to non-pathogenic types further suggests that they will be detected even in small quantities. While the detection of some (but not most) non-pathogenic types was possible in our pure-culture laboratory tests, this may be an artifact of their being the only template available. Testing for such an artifact could be done in future efforts by using *Labyrinthula* spp. “naïve” seagrass tissue (sensu [32], though even their naïve experimental shoots revealed some trace amounts) dosed with known amounts of pathogenic and non-pathogenic isolates, both singly and in various combinations. Yet, the absolute abundance of such non-pathogenic types in wild samples remains unknown. Finally, our assay consistently and reliably detected standard curve DNA concentrations that were > 0.001 cells μL^-1^, with the extraction/amplification efficiency estimate considered within the range of accepted values (e.g., 86.2% -104.0%; [38]).

To determine if qPCR is a more direct measure of pathogen presence in or on the plant host tissue, as compared to the more indirect WI method of visually assessing the areal extent of leaf surface covered by necrotic lesions [37], we compared both methods. The results indicate the two methods appear comparable to one another across a broad range of disease levels, but the correlation deteriorates at higher levels of necrosis (Fig 4). This discrepancy may be explained by the findings of Muehlstein et al. [43], who reported *Labyrinthula* sp. cells in apparently healthy, green tissue up to 5 mm from visibly blackened lesion tissue. Later studies further advanced the notion that the WI may underestimate both the number of pathogen cells present and the areal extent of damage inflicted on a region of host tissue, based on the observation that photosynthesis was significantly impaired in green eelgrass tissue beyond the local lesion sites [44]. Furthermore, “browning” could be a function of host necrosis or programmed cell death in response to pathogen infection as opposed to serving as an actual reflection of true pathogen load [58–59]. Finally, Muehlstein [8] described that *Labyrinthula* sp. cells were more abundant along the margins of disease lesions, suggesting that total areal measures of lesions may not be appropriate to quantify the degree of ‘active’ *Labyrinthula* sp. infection. Conversely, there may also be a minimum number of *Labyrinthula* sp. cells required before lesions first appear. Consequently, the results of this study contribute to the body of evidence which collectively suggests that utilizing a qPCR-based detection technique is warranted, as it can (under proper/specific conditions) reflect estimates of both prevalence and severity of the parasite-host interaction. However, modified WI estimates (e.g. based more on lesion perimeter, or lesion extent per unit time), or a combination of WI and qPCR, may still be quite useful for some applications–especially when assessing virulence (i.e. in terms of total plant damage).

### Complex immune responses driven by pathogen exposure

In order to test the commonly-held hypothesis that stress imposed by the environment reduces overall defense responses in plants, thereby increasing an individual’s susceptibility to subsequent opportunistic disease expression (in our case, necrotic lesions), we explored the competing roles of abiotic stressors, plant immunity, and pathogen exposure in our model pathosystem. Previous studies have indicated that when environmental stressors are applied in conjunction with *Labyrinthula* sp. inoculation, negative impacts on the host may be outweighed by the pathogen’s sensitivity to the same factors. For example, Bishop et al. [6] noted that elevated salinity suppressed the severity of SWD in *T*. *testudinum* individuals, and this response was attributed to the sensitivity of *Labyrinthula* sp. to hypersaline conditions. In Experiment 2, we simulated a scenario where the environmental stressor is lifted as the pathogen is introduced, in an attempt to isolate the impact of environmental stressors on the host seagrass species, and not the pathogen. This scenario is plausible, considering the high degree of stochasticity in the coastal marine environments where both species are found. Such a scenario may rest on the premise of differential response times between host and pathogen, and that the pathogen can remain present, even if relatively dormant (such as demonstrated for high temperatures; [60]). For example, hyposalinity from a tropical storm or coastal flushing events may favor a faster-growing/responding parasite as normal salinity returns.

In general, immune activity varied considerably within and among the treatment groups of Experiment 2 (Fig 5). For example, in contrast to the findings of Experiment 1, PPO activity did not vary significantly among treatment groups. In addition, activity levels were not differentially affected by the addition of the pathogen or by the presence of abiotic stressors followed by pathogen inoculation, within the context of individual treatments (Fig 5C). This observation is in contrast to previous findings, which suggest that PPO plays a role in the defense and resistance of plants against pathogen invasion [23–24]. The results detailing POX, EXOC and LYS activities are equally confounding in the context of differential activities among treatment groups. Disparate enzyme activity patterns may be an artifact of sample size limitations as supported by the emergence of significant trends when all samples were pooled together (next section, Fig 7).

### Individual variation trends

Though the findings of this exploratory study highlight the complexity of plant immune responses and the subsequent difficulty in teasing apart these factors, these results also highlight the utility of these biomarkers in more applied situations. For example, pooling immune parameter (POX, EXOC, PPO, LYS) values for each individual, similar to developing a ‘defense syndrome’ metric (sensu [61–62]), may enable detection of more robust trends that could exist between the immune metrics and pathogen load, given the high variation we see among individuals. However, correlative analyses revealed significant positive relationships between SWD severity and immune status for two of our chosen biomarkers. The results suggest that both POX and EXOC activity is upregulated as pathogen load increases in *T*. *testudinum* tissue (Fig 7A and 7B), with one interpretation implying that upregulation may have kept pathogen load from getting even higher, and that there is a cost to constraining pathogen load, relative to ambient conditions. The heat map shows the variability in response across experimental individuals at the parameters we assessed. In the most general sense, there appears to be an inverse relationship between pathogen load and LYS activity, as some of the lowest LYS values are found in individuals with the highest pathogen load and vice versa. Yet, the statistical power of this potential trend is lost in the high degree of LYS activity variation observed in individuals with moderate estimates of pathogen loading (individuals 5–18, Fig 8).

In conclusion, we successfully developed a sensitive qPCR assay that may prove universal to seagrass-pathogenic *Labyrinthula* spp. found around the world. We also developed a host immune panel that shows potential for evaluating factors that might influence constitutive levels in the field, such as more chronic stress. We also provide evidence for a pathogen load-associated immune response for two immune markers, which may help when investigating interactions involving pathogen exposure, environmental stressors, and inducible defenses in the field. In terms of the acute stresses applied here, development of SWD appears not to be a function of a compromised host unless the two induced responses (POX, EXOC) carry significant energetic costs or immune-related trade-offs. If not, the apparent opportunistic nature of some *Labyrinthula* spp. under acute host stress may be more a matter of factors favoring the pathogen (e.g. transmission and/or infection dynamics), suggesting this pathogen may often be a commensal or is taking advantage of host tolerance strategies [32, 36]. However, immune measures are always relative to what is considered ‘normal’, with the goal of preventing, limiting, or eliminating infections. This highlights the challenge of elucidating potential mechanisms that shift opportunistic microbe-host relations, as a compromised host is often the default assumption. Finally, even the non-induced markers should be useful, as their constitutive levels may involve trade-offs dependent on environmental factors other than pathogen exposure. Thus, the methodology presented here broadens the options available for identifying possible SWD defense strategies that might eventually provide evolutionary insights (e.g., [63]) into the greater seagrass-*Labyrinthula* spp. pathosystem.

## Supporting information

S1 Table Summary statistics of one-way ANOVA results describing global treatment effects in immune biomarker assays (peroxidase, exochitinase, polyphenol oxidase, and lysozyme) from Experiment 1. (TIFF)

S2 Table Results of multiple comparisons LSD post hoc tests (p-values) of immune biomarker assays (parts A-D: Peroxidase, exochitinase, polyphenol oxidase, and lysozyme). Pairwise comparisons between treatment groups [ambient; hypersalinity; hyposalinity; elevated temperature] from Experiment 1 are shown. Asterisk (*) represents two-tailed significance at α = 0.05. Insignificant differences between treatments are shaded in light gray; redundant comparisons are shaded in dark gray.
(TIFF)

S3 Table Raw disease severity (i.e. Wasting Index: Percent leaf area of lesion extent) and pathogen load (cell counts of *Labyrinthula* sp. “E” isolate 8b obtained through qPCR) measures, by abiotic treatment from Experiment 2, used in the AICc assessment (Fig 4). (TIFF)

S4 Table Summary statistics of one-way ANOVA results describing global treatment effects in immune biomarker assays (peroxidase, exochitinase, polyphenol oxidase, and lysozyme) from Experiment 2. (TIFF)

S5 Table Results of multiple comparisons LSD post hoc tests (p-values) of immune biomarker assays (parts A-D: Peroxidase, exochitinase, polyphenol oxidase, and lysozyme) from Experiment 2. Pairwise comparisons between treatment groups [ambient (-) *Laby*.; ambient (+) *Laby*.; hypersalinity (+) *Laby*.; hyposalinity (+) *Laby*.; elevated temperature (+) *Laby*.] are shown. One and two asterisks (*, **) represent two-tailed significance at α = 0.05 and α = 0.01, respectively. Insignificant differences between treatments are shaded in light gray; redundant comparisons are shaded in dark gray.
(TIFF)

S6 Table Results of multiple comparisons LSD post hoc tests (p-values) comparing pathogen load (*Labyrinthula* cells per mg dry weight of leaf tissue) with treatment. Pairwise comparisons between treatment groups [control/ambient (-) *Laby*.; ambient (+) *Laby*.; hypersalinity (+) *Laby*.; hyposalinity (+) *Laby*.; elevated temperature (+) *Laby*.] from Experiment 2 are shown. One and two asterisks (*, **) represent two-tailed significance at α = 0.05 and α = 0.01, respectively. Insignificant differences between treatments are shaded in light gray; redundant comparisons are shaded in dark gray.
(TIFF)

S7 Table Summary statistics of one-way ANOVA results describing global treatment effects on pathogen load (*Labyrinthula* sp. “E” isolate 8b cells per mg dry weight of leaf tissue) from Experiment 2, before (“All Treatments”) and after removal of the “Ambient (-) *Laby*. treatment”. Double asterisks (**) represent two-tailed significance at α = 0.01.
(TIFF)

S8 Table One-way ANOVA results detailing global treatment effects on pathogen load (*Labyrinthula* sp. cells per mg dry weight of leaf tissue) in Experiment 2, after removal of the Ambient (-) *Laby*. treatment from the analysis. (TIFF)
