# Supplementary figures and images for: Integrating host immune status, Labyrinthula spp. load and environmental stress in a seagrass pathosystem: Assessing immune markers and scope of a new qPCR primer set
Source: PLoS One. 2020 Mar 13;15(3):e0230108. doi: 10.1371/journal.pone.0230108 (PMC7069685; doi:10.1371/journal.pone.0230108)

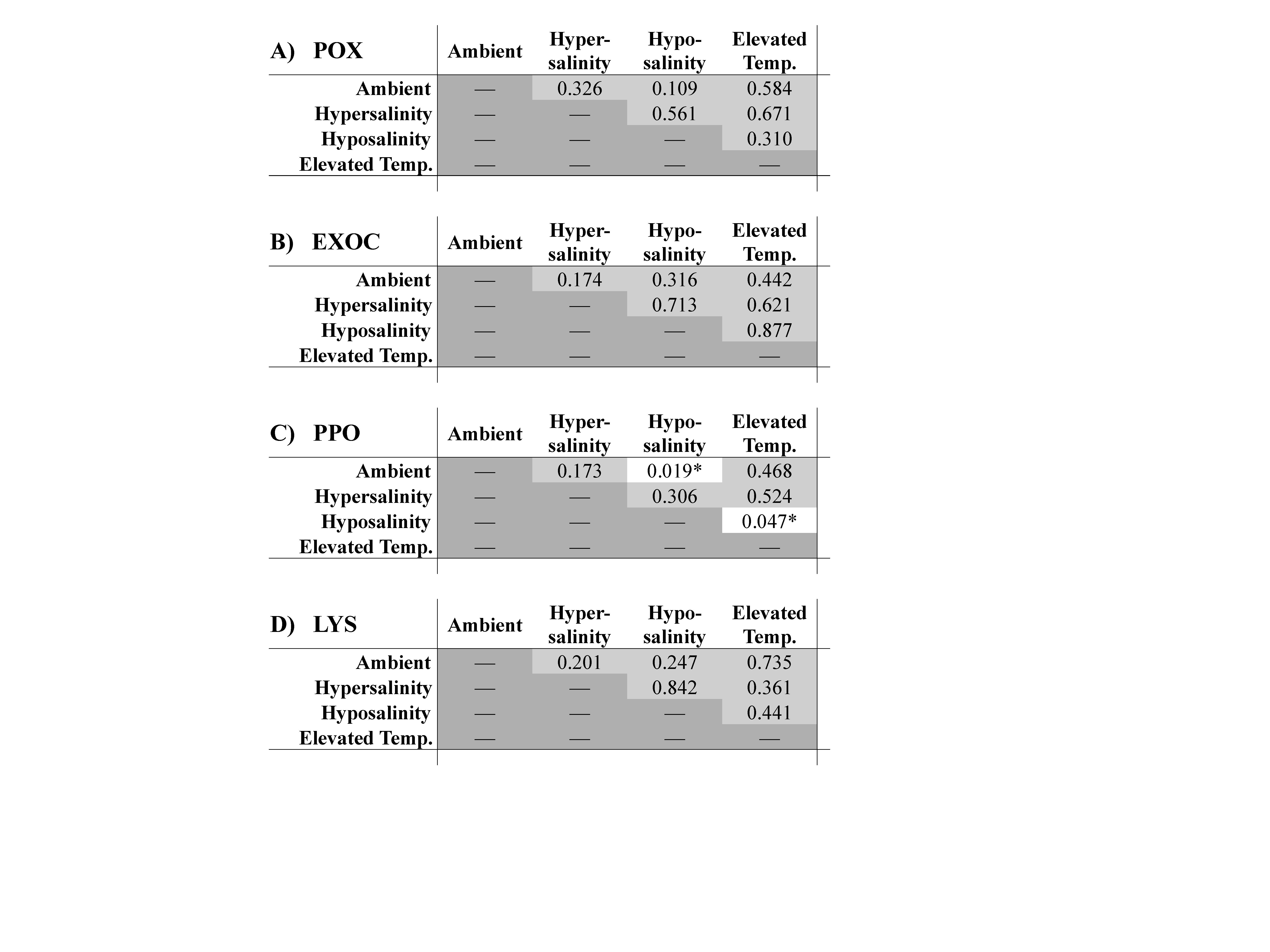

Supplement: S2 Table — Pairwise comparisons between treatment groups [ambient; hypersalinity; hyposalinity; elevated temperature] from Experiment 1 are shown. Asterisk (*) represents two-tailed significance at α = 0.05. Insignificant differences between treatments are shaded in light gray; redundant comparisons are shaded in dark gray. (TIFF) [file pone.0230108.s002.tiff]

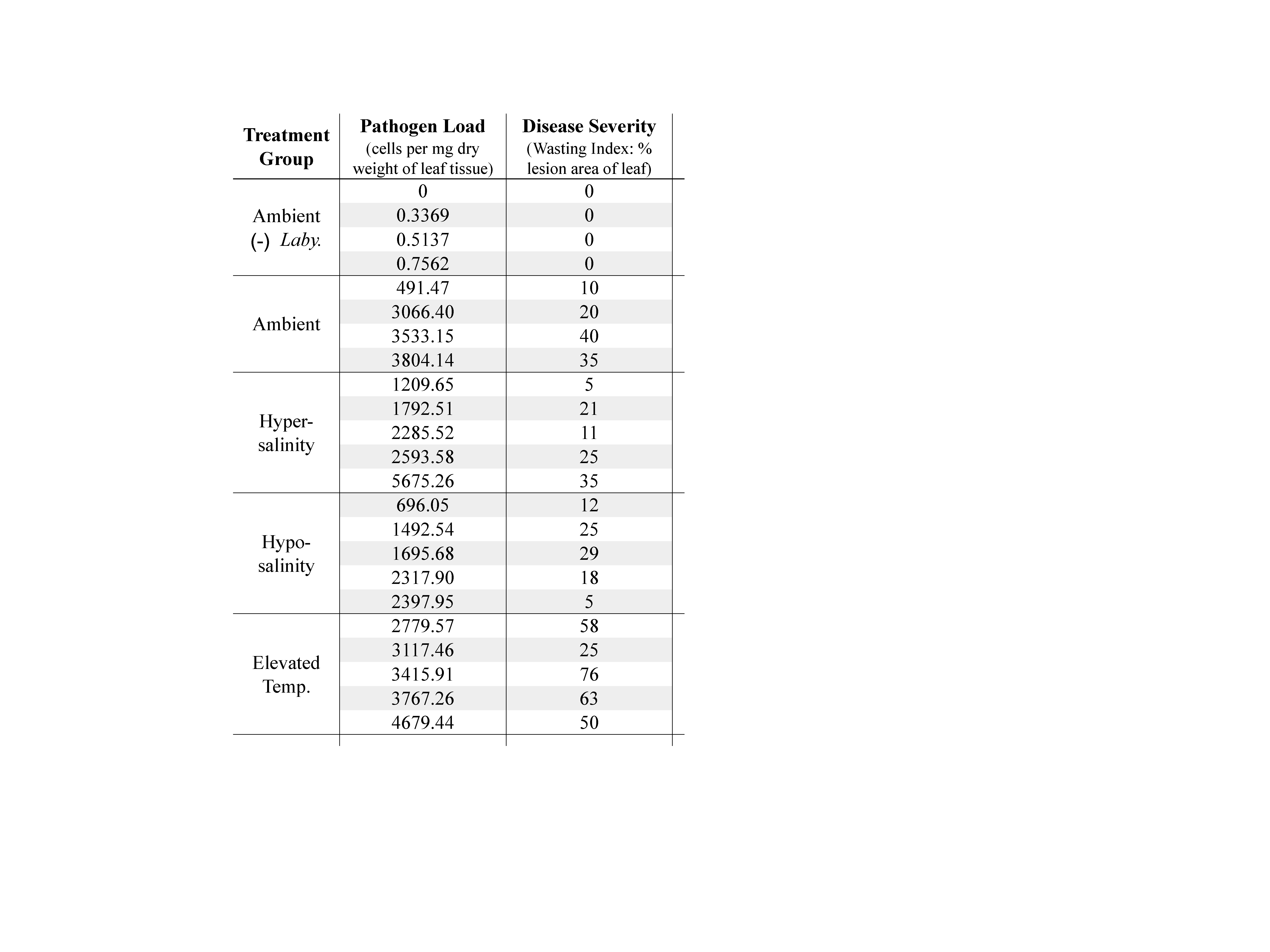

Supplement: S3 Table — (TIFF) [file pone.0230108.s003.tiff]

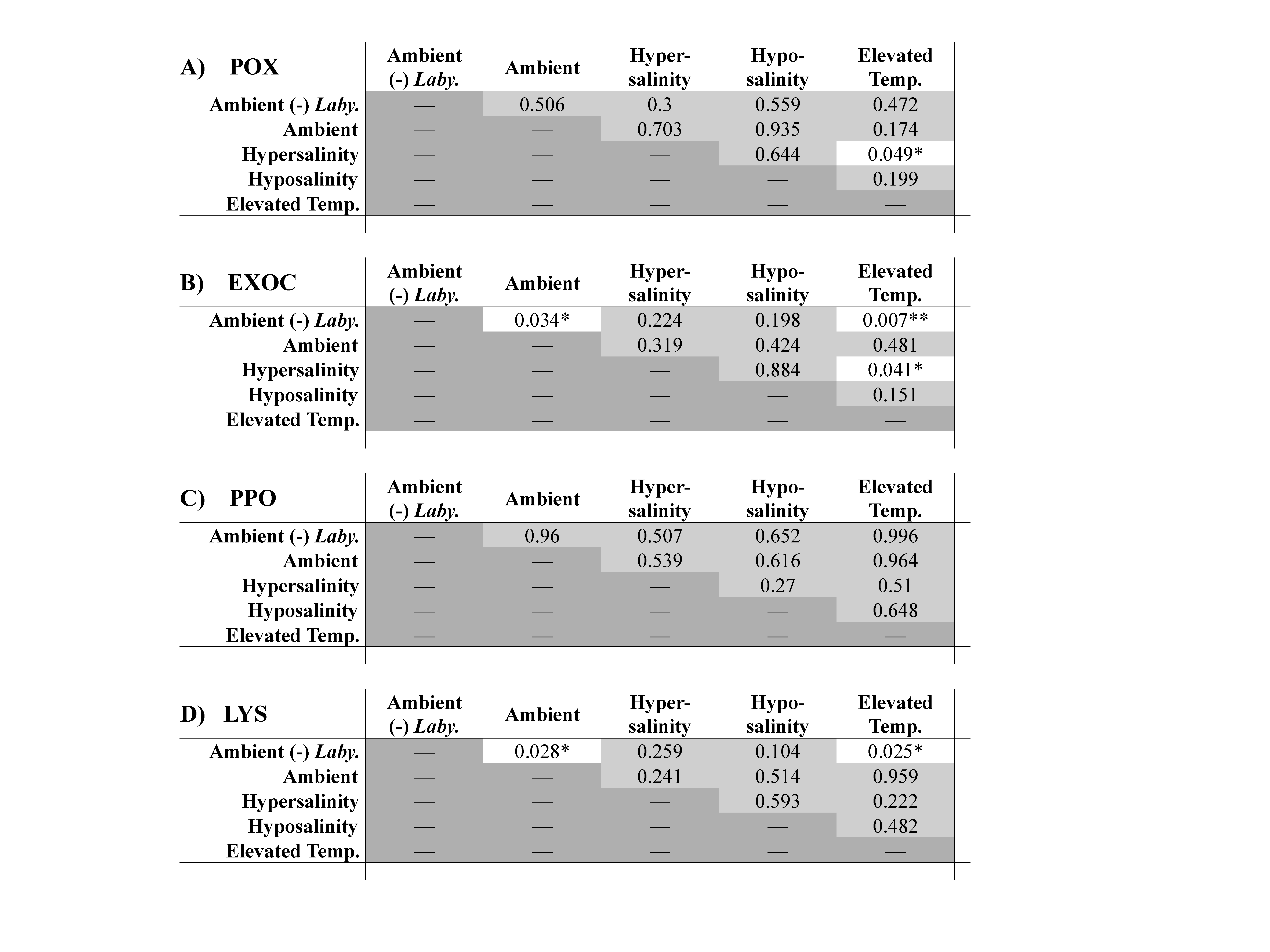

Supplement: S5 Table — Pairwise comparisons between treatment groups [ambient (-) Laby.; ambient (+) Laby.; hypersalinity (+) Laby.; hyposalinity (+) Laby.; elevated temperature (+) Laby.] are shown. One and two asterisks (*, **) represent two-tailed significance at α = 0.05 and α = 0.01, respectively. Insignificant differences between treatments are shaded in light gray; redundant comparisons are shaded in dark gray. (TIFF) [file pone.0230108.s005.tiff]

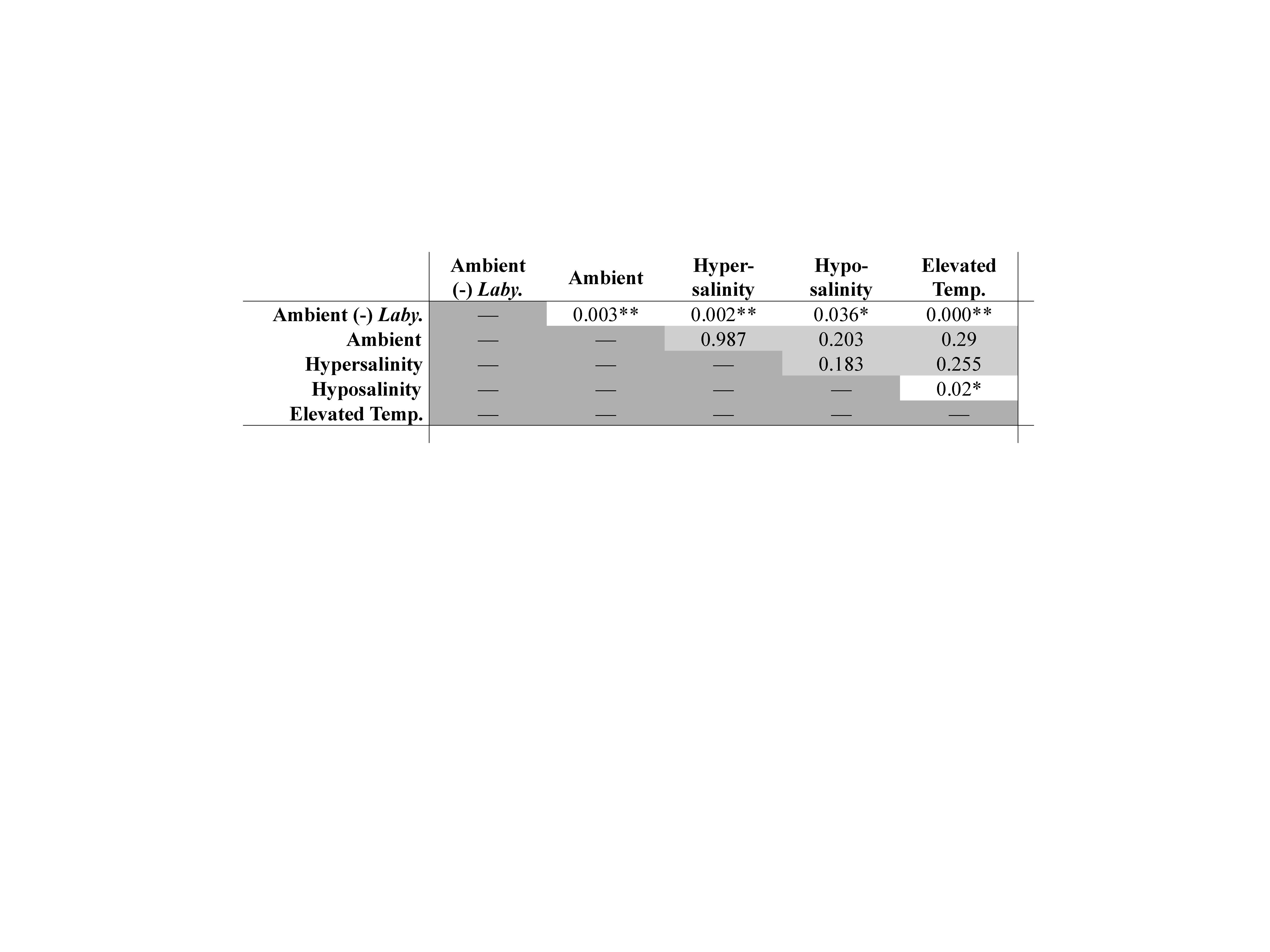

Supplement: S6 Table — One and two asterisks (*, **) represent two-tailed significance at α = 0.05 and α = 0.01, respectively. Insignificant differences between treatments are shaded in light gray; redundant comparisons are shaded in dark gray. (TIFF) [file pone.0230108.s006.tiff]

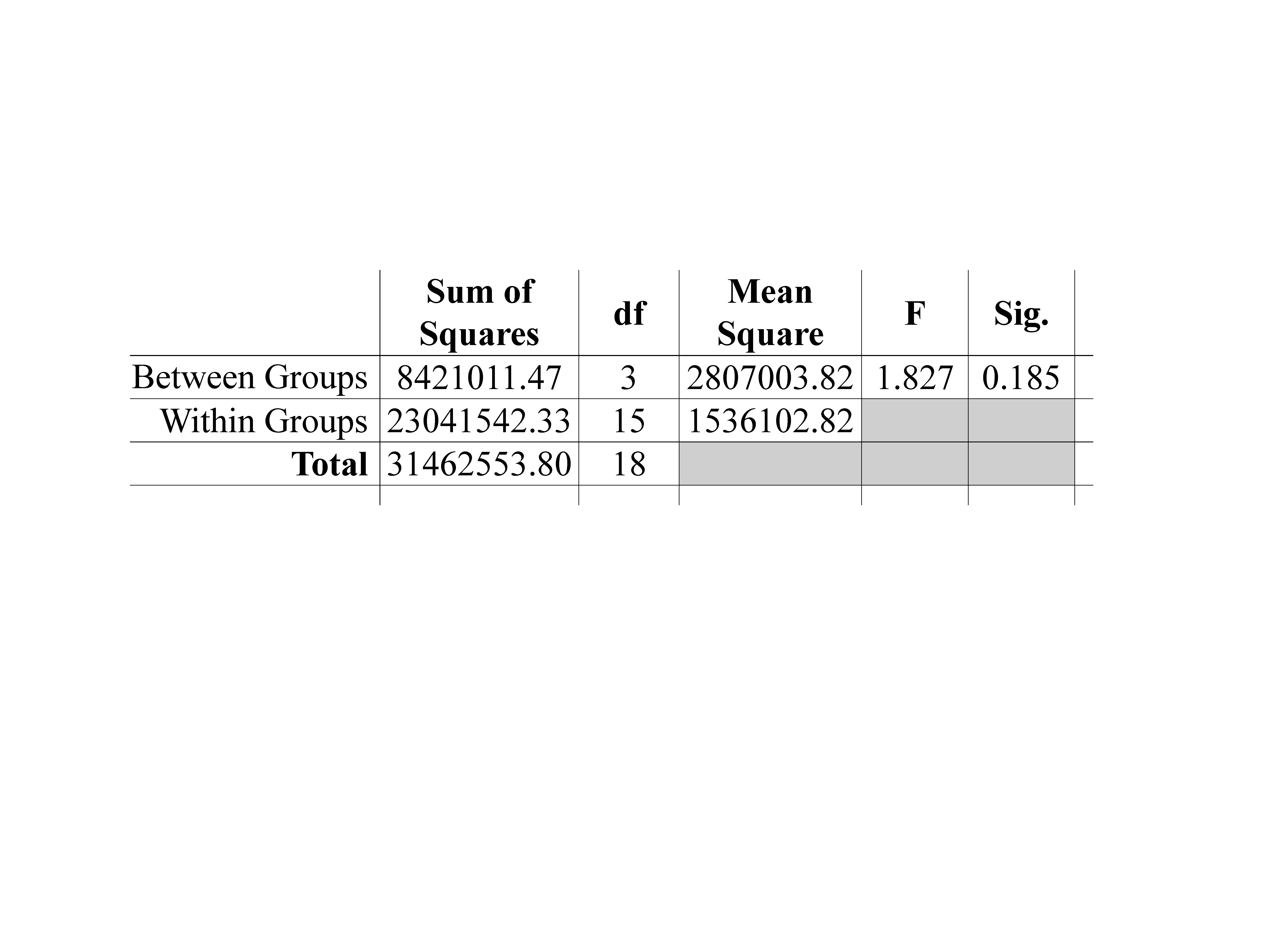

Supplement: S8 Table — (TIFF) [file pone.0230108.s008.tiff]
